# Bacterial Genotoxins: Merging the DNA Damage Response into Infection Biology

**DOI:** 10.3390/biom5031762

**Published:** 2015-08-11

**Authors:** Francesca Grasso, Teresa Frisan

**Affiliations:** Department Cell and Molecular Biology, Karolinska Institutet, S-171 77 Stockholm, Sweden; E-Mail: francesca.grasso@ki.se

**Keywords:** bacterial genotoxins, DNA damage response, cancer, chronic infection, probiotics

## Abstract

Bacterial genotoxins are unique among bacterial toxins as their molecular target is DNA. The consequence of intoxication or infection is induction of DNA breaks that, if not properly repaired, results in irreversible cell cycle arrest (senescence) or death of the target cells. At present, only three bacterial genotoxins have been identified. Two are protein toxins: the cytolethal distending toxin (CDT) family produced by a number of Gram-negative bacteria and the typhoid toxin produced by *Salmonella enterica* serovar Typhi. The third member, colibactin, is a peptide-polyketide genotoxin, produced by strains belonging to the phylogenetic group B2 of *Escherichia coli*. This review will present the cellular effects of acute and chronic intoxication or infection with the genotoxins-producing bacteria. The carcinogenic properties and the role of these effectors in the context of the host-microbe interaction will be discussed. We will further highlight the open questions that remain to be solved regarding the biology of this unusual family of bacterial toxins.

## 1. Introduction

Cells of our body are exposed to several physical (UV, X-rays) and numerous chemical agents that promote DNA damage. The latter includes reactive oxygen species (ROS) produced by our own cellular metabolism or as a consequence of acute and chronic infections [1]. To this long list, we need to add a new category of effectors, namely bacterial genotoxins.

The first bacterial toxin shown to induce DNA single strand (SSBs) and double strand (DSBs) breaks is the family of the cytolethal distending toxins (CDTs) [2,3], which are produced by several Gram negative bacteria, such as *Escherichia coli*, *Aggregatibacter actinomycetemcomitans*, *Haemophilus ducreyi*, *Shigella dysenteriae*, *Campylobacter* sp., and *Helicobacter* sp., (reviewed in [4]). CDT was initially discovered in pathogenic *E. coli* strains as an effector that induces a remarkable cell distension, evident 120 h after addition of bacterial culture supernatants to cultured cells, hence the name of the toxin, leading eventually to cell death [5].

In the wake of this puzzling discovery, two more bacterial effectors with genotoxic activity have been characterized: the typhoid toxin produced by *Salmonella enterica* serovar Typhi (*S.* Typhi) [6], and colibactin, encoded within the *pks* genomic island, present in *E. coli* strains of the phylogenetic group B2 [7].

This review will focus on the effects of these toxins on mammalian cells. We will also present recent findings demonstrating that the proteins ExoS from *Pseudomonas aeruginosa* [8] and Usp from uropathogenic strains of *Escherichia coli* [9], and a still non-characterised effector(s) from *Helicobacter pylori* [10] promote DNA damage in a ROS-independent manner.

The role of these effectors in the context of bacterial-associated carcinogenesis and infection/colonization will be discussed.

## 2. CDT and Typhoid Toxin

### 2.1. Structure

**Figure 1:**
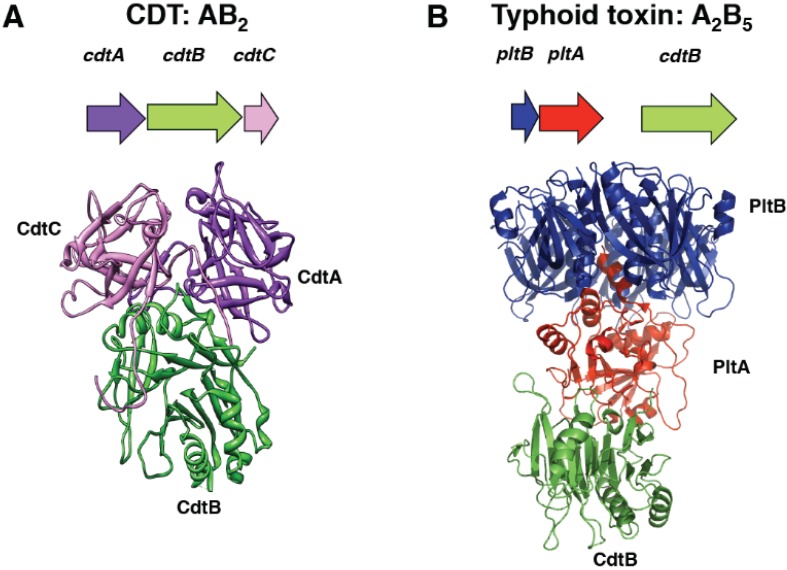
CDT (cytolethal distending toxin) and typhoid toxin structure (**A**) Schematic representation of the CDT genes from *H. ducreyi* and the crystal structure of the holotoxin, adapted from Nesic *et al.* [11], PDB access number: 1SR4. The CdtB is the active subunit, possessing DNase activity, while the CdtA and CdtC accessory subunits constitute the binding component of this AB_2_ toxin; (**B**) Schematic representation of the typhoid toxin genes from *Salmonella enterica* serovar Typhi and crystal structure of the holotoxin, adapted from Song *et al.*, PDB access number: 4K6L [16]. CdtB is connected by PltA to a pentameric disc made by five PltB monomers (the “B” moiety). This A_2_B_5_ toxin contains two active subunits: CdtB, homologous to mammalian DNase I, a characteristic shared with CDTs, and the ADP ribosyl transferase PltA.

CDTs and the typhoid toxin are AB toxins, where “A” stands for active subunit and “B” for binding moiety, which is required for the internalization of the “A” component into the target cell. The crystal structure of these two toxins is very different. The members of the CDT family are AB_2_ trimers [11], composed by the active subunit CdtB, and two binding moieties: CdtA and CdtC (Figure 1A). The three components of the holotoxin are encoded by a single operon (Figure 1A) [12].

The sequence homology between the *cdtA*, *cdtB*, and *cdtC* genes among different bacteria species that produce this toxin is variable, and several CDTs have been identified even within the same species (e.g., *E. coli*). The most conserved gene is the *cdtB*, encoding for the active subunit, while the genes encoding for the CdtA and CdtC subunits are the most divergent [13]. To avoid confusion among different CDTs, a specific nomenclature has been proposed by Cortes-Bratti *et al.* [14] and Jinadasa *et al.* [15]. Each member is specified by defining the producing bacterium using the first capitalized letter of the genus followed by the first three letters of the species name in lower case before CDT and, if necessary, the strain number or other common designation after CDT (e.g., EcolCDT-I for the CDT I variant produced by *E. coli*, or HducCDT for the toxin produced by *H. ducreyi*) [14,15].

The typhoid toxin is encoded by three genes: *pltB*, *pltA*, and *cdtB*, whose products are organised into a A_2_B_5_ complex [16], where CdtB and PltA are the “A” subunits, and the pentameric ring formed by PltB represents the binding “B” unit (Figure 1B).

In spite of the overall diversity, these toxins shared a common feature, the CdtB subunit, which is functionally and structurally homologous to the mammalian DNase I [11,16]. This enzyme can cleave DNA either in the form of a naked plasmid [2] or as highly organized eukaryotic DNA [3,17,18]. The typhoid toxin has an additional “A” subunit (PltA) (Figure 1B), which is an ADP ribosyl transferase, and whose cellular targets have not been yet identified [16].

### 2.2. Activity

Most of the studies on the activity of CdtB have been performed with CDTs, since the typhoid toxin has been identified and characterized as a genotoxin only recently [6]. One striking feature of the DNase activity of CdtB is its reduced potency when compared to the mammalian DNase I. *In vitro* experiments estimated that the specific DNA-nicking activity of *E. coli* CdtB is 100 times lower than that of purified human or bovine DNase I [19]. In HeLa cells, microinjection of purified bovine DNase I causes profound changes in the chromatin structure at a concentration of 4–40 pg/mL, while a much higher amount of purified CdtB from *H. ducreyi* (40 μg/mL) is necessary to obtain asimilar effect [20].

These data have been quite puzzling in the field, and the key question is why a bacterial genotoxin with such a low efficacy, has been maintained, and possibly horizontally transferred, in several species? A possible answer may come from studies assessing the role of these effectors in *in vivo* infection, and will be discussed later.

The possibility of horizontal transfer has been suggested by the demonstration that the genes encoding for several members of the *E. coli* CDTs are flanked by genetic mobile elements reminiscent of lambdoid prophages [21,22], or by P2-like phage sequences [23], and the EcolCDT-III operon is located within the pVir conjugative plasmid [24].

Regarding the type of DNA damage induced by CDTs, a detailed kinetic analysis has been performed by Fedor and colleagues who demonstrated that low doses of the *E. coli* CDT-I (50 pg/mL) induce SSBs from 3 h–6 h post-intoxication. These lesions are further converted into DSBs during the S phase of the cell cycle due to inhibition of the progression of the replicative fork as a consequence of unrepaired SSBs [25]. Conversely, higher toxin doses (above 75 ng/mL) induce mainly DSBs, possibly due to the induction of juxtaposing SSBs on opposite strands. This latter effect is independent of the cell cycle phase. These observations reconcile some discrepancies in the literature, where transit through the S phase of the cell cycle was demonstrated to be required for the activity of EcolCDT [26,27], while other authors demonstrated that the *H. ducreyi* toxin induces DNA damage also in non proliferating cells [20].

Based on the low CdtB efficacy as DNase, it was suggested that this subunit might have an additional enzymatic activity [28]. This possibility was supported by the observation that DNase I belongs to a broad superfamily that includes nucleases as well as various Mg^2+^-dependent phosphoesterases such as the inositol polyphosphate 5-phosphatase [28]. Shenker and colleagues have tested this hypothesis and reported that the CdtB subunit from *A. actinomycetemcomitans* exhibits PI-3,4,5-triphosphate (PI-3,4,5-P_3_) phosphatase activity *in vitro* [29]. Furthermore, intoxication significantly reduces the levels of PI-3,4,5-P_3_ in the human T cell lines Jurkat, CEM, and Molt, which carry mutations in the *PTEN* and/or *SHIP1* genes and consequently present high intracellular levels of PI-3,4,5-P_3_ [29]. The toxin activity correlates with induction of cell cycle arrest, the classical effect observed for CDT and typhoid toxin intoxication [4,6]. Interestingly, the T cell line HUT78, which contains functional levels of PTEN and SHIP1 and low levels of PI-3,4,5-P_3_, does not show any sign of cell cycle arrest [29]. However, the authors did not assess whether the CDT treatment in these experimental conditions induced phosphorylation of histone H2AX [29], the classical sign of DNA damage [30].

These data suggest that the phosphatase activity of AactCDT becomes relevant under certain conditions (e.g., high intracellular levels of PI-3,4,5-P_3_,), while the genotoxic effect of CDTs has been observed in all the cell types tested, both established cell lines as well as primary cells, the only limitation being the species specificity barrier [4,15].

### 2.3. Internalization and Nuclear Translocation

Among all bacterial AB toxins, CDTs and the typhoid toxin are the only effectors that need to be translocated into the nuclear compartment in order to interact with their target: the cellular DNA. Here we will briefly review the internalization pathway and refer to other reviews for a more detailed analysis [31].

Most of the studies aimed at characterizing the internalization pathways have been performed by using recombinant purified CDT, and mainly EcolCDT-II, EcolCDT-III, AactCDT, CjejCDT and HducCDT. The results are not completely overlapping, indicating that, depending on the specific toxin, there are different surface receptors and divergent intracellular pathways. However, these toxins share a common feature: the retrograde transport from the plasma membrane through the endosomal compartment, the trans-Golgi network (TGN), and the endoplasmic reticulum (ER) [32,33,34,35,36,37,38,39,40]. It is still unclear how the toxin is further translocated from the ER to the nuclear compartment. Several lines of evidence suggest that the toxin may exploit transit through an alternative ER-associated degradation (ERAD) pathway, which relays on translocation of misfolded proteins through specialized transporters.

Indeed, three components of the ERAD pathway, Derlin-2, the E3 ubiquitin ligase Hrd1, and the AAA ATPase 97 (p97), have been identified as essential for the intoxication mediated by Aact-, Hduc-, Cjej-, and EcolCDT-III [41]. However, the Derlin-2 WR domain and the direct Derlin-2-p97 interaction, known to be essential for transport of misfolded proteins, are dispensable for CDT translocation, suggesting that members of this toxin family do not follow the classical ERAD pathway. This is in line with previous observations showing that the HducCdtB is a poor substrate for ERAD because it is highly stable [42], and overexpression of dominant negative mutant forms of Derlin-1 and Derlin-2, which abolish translocation of misfolded proteins, failed to prevent translocation of HducCDT [37].

Much less data are available regarding the internalization and translocation pathway of the typhoid toxin. Using a recombinant purified toxin, Song and co-workers have identified Podocalyxin-like protein 1 (PODXL) and CD45 as the toxin receptor on the human epithelial cell line Henle-407 and on cell lines of hematopoietic origin, respectively [16]. These data indicate that the identity of the receptor is cell type specific, and may explain the broad range of cell types that are sensitive to the typhoid toxin. However, an interesting issue in understanding the delivery of the typhoid toxin to the nucleus of the target cells comes from the observation that this toxin is expressed by *S.* Typhi only when the bacterium is internalized and replicates within a specialised vacuole, known as Salmonella-containing vacuole (SCV), in the host cell [6,43]. Thus, this effector has to cross several membrane compartments before gaining access to the cellular DNA. The toxin can be released from the bacterium within outer membrane vesicles (OMVs), where it is protected from host proteases, such as trypsin [44]. These vesicles are then released from the infected cells into the extracellular environment, where up to 30% of the total extracellular vesicles have the characteristics of bacterial secreted OMVs [44]. This process required an intact SCV, suggesting that the toxin-loaded OMVs are shed within this vacuolar compartment, and subsequently packed into larger cargos that detach from the SCV, relaying on anterograde transport toward the cellular cortex on the microtubule and actin cytoskeleton to be released outside the infected cells [44]. The secreted toxin-loaded OMVs, can be further internalized by the bystander non-infected cells in a dynamin-dependent manner. The toxin is retrogradely translocated through the TGN, before entering the nuclear compartment and promoting DNA damage [44]. This data indicate that OMVs may represent an efficient way to deliver a concentrated amount of bacterial effectors at distant locations in cargos protected by cellular proteases. Toxin release from cells and infection of bystander cells was also demonstrated by Spano and colleagues in epithelial cells infected with *S.* Typhi [43].

The typhoid toxin can also promote DNA damage within the infected cells, and it is interesting to assess how this pool of toxin is translocated to the nuclear compartment. Preliminary data performed in our laboratory suggest that the translocation pathway of the toxin within the infected cell is different from that used by the toxin secreted into the extracellular environment (Frisan *et al.* unpublished observation). At this stage it is still not clear whether the different trafficking routes are related to the presence of the toxin within OMVs, secreted into the extracellular environment, *versus* possible release of free toxin in the cytoplasm that may reach the nucleus of the infected cells by direct translocation through the nuclear pores.

### 2.4. Cellular Responses to CDTs and Typhoid Toxin

**Figure 2:**
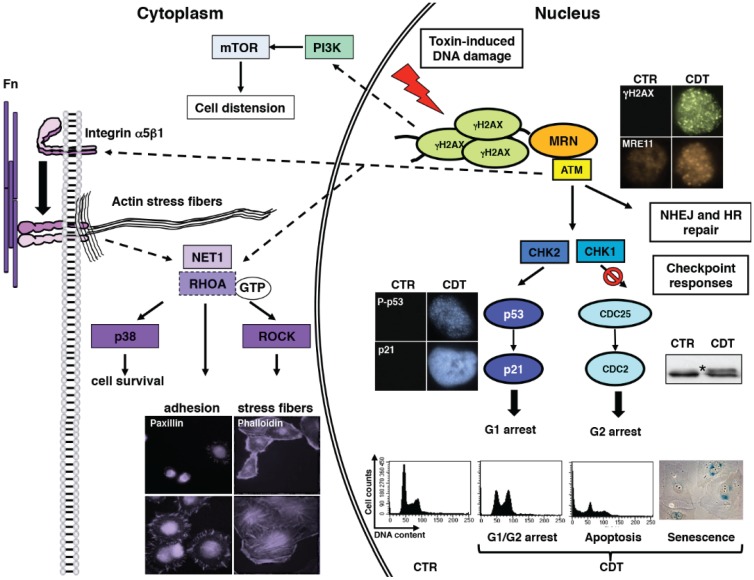
Activation of DNA damage response in CDT-intoxicated cells. Upon intoxication, the MRN complex (visualized by fluorescence microscopy using a MRE11 specific antibody) is recruited and promotes full activation of ATM kinase at the site of the damage. This process results in: (i) phosphorylation of histone H2AX (γH2AX) (visualized by fluorescence microscopy using a phospho-H2AX specific antibody); (ii) phosphorylation and stabilization of the tumor suppressor p53 (visualized by fluorescence microscopy using an antibody specific for the phosphorylated form of p53 on Ser15) and increased expression of its target gene p21 (visualized by fluorescence microscopy using a p21 specific antibody); and (iii) activation of the kinases CHK2 and CHK1, leading to inactivation of the CDC25 phosphatase, and consequent hyperphosphorylation and inactivation of the cyclin dependent kinase CDC2 (marked with an asterisk in representative western blot in the figure). The results of the checkpoint responses will lead to cell cycle arrest either in the G1 or G2 phases of the cell cycle (assessed by the propidium iodide staining) to allow repair. If the damage is beyond repair, this response will result in activation of the tumorigenesis barrier, which will eliminate the altered cell by apoptosis (assessed by accumulation of cells in the subG1 phase of the cell cycle by propidium iodide staining) or induction of cellular senescence (visualized by the detection of the senescence-associated beta-galactosidase activity). The ATM-dependent signalling is also transduced in the cytosol, promoting a conformational change of integrin β1 and activation of the small GTPase RHOA. This process results in: (i) enhanced formation of focal adhesion (visualized by Paxillin staining) and cell spread; (ii) induction of actin stress fibers (visualized by phalloidin staining); and (iii) activation of p38 MAPK-survival signals. The characteristic distension observed in epithelial cells exposed to CDT is dependent on activation of the PI3K and its downstream effector mTOR.

Upon delivery of the CdtB to the nucleus of the target cells, the toxin induces DNA SSBs and, mostly, DSBs, and activates the classical DNA damage response (DDR), which resembles the response to ionizing radiation, a well-characterised genotoxic agent [45], resulting in cell cycle arrest or cell death depending on the cell type or on the toxin dose used [4]. CDTs produced by different bacteria induce similar effects on the target cells, indicating that once the DNA is damaged, the cell will activate the default DDR, as summarised in Figure 2. Thus, as expected, the initial response to the intoxication is partly ATM-dependent, since a delayed response has been detected in lymphoblastoid cells derived from Ataxia telangiectasia (AT)-patients, carrying a non-functional kinase [45]. Early CDT intoxication or infection with typhoid toxin-producing *Salmonella* is associated with: (i) phosphorylation of Histone 2AX (γH2AX), which is currently used as a standard method to detect bacterial genotoxin-induced DNA damage; (ii) re-localization of the DNA repair proteins, such as MRE11, NBS1, and RAD50 at the sites of the damaged DNA; (iii) activation of the tumor suppressor p53 and its transcriptional target, the cyclin-dependent kinase inhibitor p21; (iv) phosphorylation of the checkpoint kinases CHK1 and CHK2; and (v) inactivation of CDC25 phosphatase and accumulation of the inactive hyper-phosphorylated form of CDC2 [20,27,43,44,45,46,47,48,49,50,51,52].

The immediate consequence of the intoxication-induced DDR is to block the cell cycle progression in the G1 and/or G2 phases [45,48] and initiate DNA repair. Experimental evidences indicate that both Homologous Recombination (HR) and Non Homologous End Joining (NHEJ) repair systems are activated in response to the intoxication. Indeed, components of the HR pathway, such as the orthologous of the mammalian MRN complex, Rad51, and Rad55, have been identified as essential genes to sustain cell viability in *Saccharomyces cerevisiae* upon induction of DNA damage by CjejCdtB [53]. This observation was confirmed in the mammalian system by two independent studies. Fedor *et al.* demonstrated that siRNA knockdown of RAD51 in HeLa cells strongly promotes cell death upon exposure to EcolCDT [25], and Fahrer *et al.*, using cell lines deficient for each of the repair systems, provided evidence that both HR and NHEJ protects cells from DNA lesions induced by HducCDT [52].

In most of the published studies, the final outcome of intoxication is failure to repair the damage, resulting in either cell death or a long-term cell cycle arrest state known as cellular senescence [4,54]). Interestingly, there is a cell type-dependency regarding the choice between senescence and apoptosis. Cells of epithelial and mesenchymal lineage mainly undergo senescence, characterised by persistently activated DNA damage signalling (detected as 53BP1/γH2AX-positive foci), enhanced senescence-associated β-galactosidase activity, expansion of promyelocytic leukemia (PML) nuclear bodies, and expression of IL-6, IL-8, IL-20, and IL-24 [55]. Conversely, cells of haematopoietic lineage are more susceptible to apoptosis upon intoxication. Indeed, treatment of activated human T lymphocytes or the T cell leukemia lines Jurkat and MOLT-4 with AactCDT induces caspase-dependent cell death [56,57]. Ohara and co-workers further showed that a subpopulation of Jurkat cells dies at a later stage of intoxication (more than 24 h post-intoxication) by a caspase-independent process, possibly due to accumulation of ROS [58].

**Figure 3:**
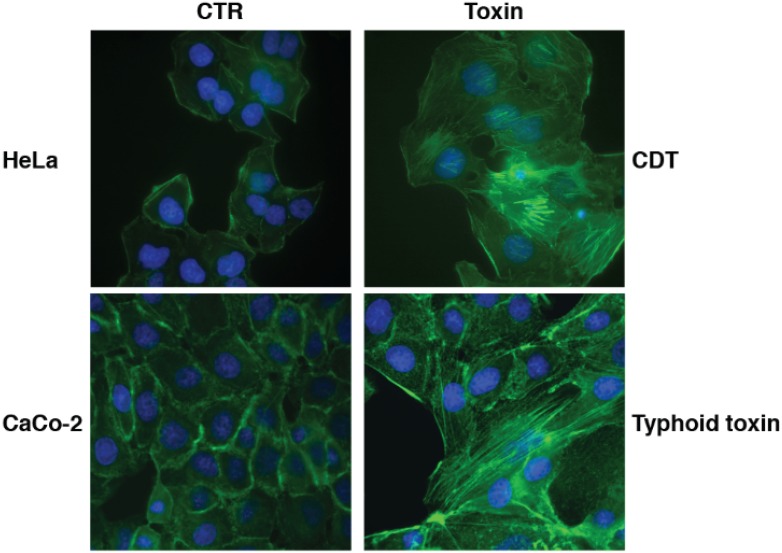
CDT and typhoid toxin promote cell distension and formation of actin stress fibers. HeLa cells were either left untreated (CTR) or exposed to HducCDT (Toxin, 2 μg/mL) for 24 h. CaCo-2 cells were infected with a strain of *Salmonella enterica*, serovar Typhimurium, expressing an active (Toxin) or a mutant (CTR) typhoid toxin at a multiplicity of infection of 25:1, and further incubated for 24 h, as described in [44]. Polymerized actin was visualized by staining with FITC-phalloidin (green), and nuclei were counter-stained with Hoechst 33258 (blue).

This dichotomy may depend on activation of survival signalling pathways in adherent cells, which may prevent apoptosis and lead to the acquisition of the senescent phenotype. A profound cell distension and formation of actin stress fibers has been observed in epithelial and mesenchymal cells exposed to CDT, infected with the typhoid toxin-producing *Salmonella*, or exposed to ionizing radiation [6,18,46,59,60] (Figure 3). The cell distension requires a functional PI3-kinase and its downstream effector mTOR, while re-organization of the actin cytoskeleton is regulated by the ATM-dependent activation of the small GTPase RHOA (Figure 2), which also promotes cell survival, via the mitogen-activated protein kinase (MAPK) p38 and its downstream target MAPK-activated protein kinase 2 [18,61]. RHOA activation has not been detected in intoxicated lymphoblastoid B cell lines, cells of hematopoietic origin [18], which undergo apoptosis upon intoxication [45]. Thus, this signalling pathway may represent one of the mechanisms that regulate the choice between senescence *versus* apoptosis in response to genotoxic stress.

Activation of survival signals may not only regulate the decision between senescence *versus* apoptosis in case of induction of DNA damage, which is beyond repair, but may have more serious consequences when, due to accumulation of genomic instability, the proper activation of the checkpoint and senescence responses is altered, leading to survival and growth of cells that have the potential of progressing towards a malignant phenotype. This issue will be discussed in more detail in the next sections.

The ATM-dependent signal induced by CDT or typhoid toxin in adherent cells is also associated with an inside-out activation of integrin β1, which results in enhanced cell adhesion and spread, increased formation of focal adhesions, and prolonged survival of the intoxicated cells in anchorage- independent conditions [60] (Figure 2). This nuclear-dependent activation of integrin signalling was not previously reported in the literature, indicating that bacterial genotoxins are useful tools to identify novel cellular signalling pathways.

## 3. Colibactin

**Figure 4:**
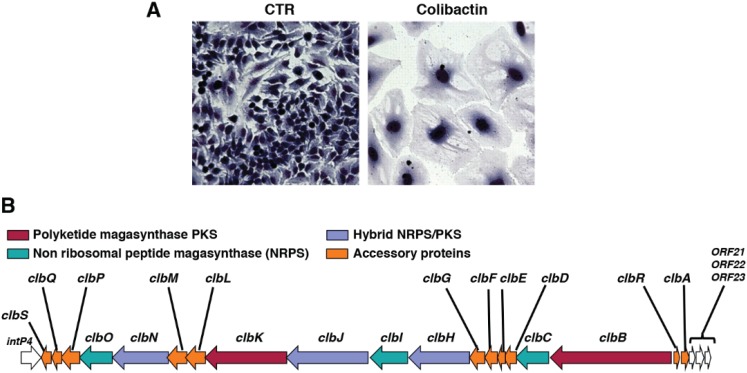
Effects of colibactin on mammalian cells and its genomic organization (**A**) HeLa cells were infected with the *E. coli* strain IHE3034 carrying the *pks* genomic island for 4 h. Cells were washed and further incubated for 72 h with gentamicin before staining with Giemsa (adapted from [Nougayrede, J.P.; Homburg, S.; Taieb, F.; Boury, M.; Brzuszkiewicz, E.; Gottschalk, G.; Buchrieser, C.; Hacker, J.; Dobrindt, U.; Oswald, E. *Escherichia coli* induces DNA double-strand breaks in eukaryotic cells. *Science* 2006, *313*, 848–851]. Reprinted with permission from AAAS); (**B**) Schematic representation of the *pks* genomic island that encodesthe enzymes and accessory proteins required for synthesis of an active colibactin in the *E. coli* strain Nissle 1917, adapted from Homburg *et al.* [67] with permission from the authors.

Colibactin is the second bacterial genotoxin, identified in 2006, when infection of epithelial cells with *E. coli* strains of the phylogenetic group B2 was shown to promote cell cycle arrest and a progressive enlargement of the cell body and nucleus [7]: a phenotype that was reminiscent of the effect of CDTs (Figure 4A). Differently from CDTs, this activity was not induced by a protein effector, but was associated with the presence of a 54 Kb genomic island, located in the *asnW* tRNA locus, an integration hotspot for foreign mobile DNA elements [62]. This island is referred to as the *pks* island [7], and contains 23 putative open reading frames (ORFs), including three nonribosomal peptide megasynthetases (NRPS), three polyketide megasynthetases (PKS), two hybrid NRPS/PKS megasynthetases, and ten accessory, tailoring, and editing enzymes (Figure 4B). Mutation analysis demonstrated that all the PKS and NRPS and eight of the accessory genes are required for production of an active genotoxin, indicating that the toxicity is due to a polyketide-peptide hybrid cytotoxin that was defined as colibactin [7].

The cytopathic effect is dependent on live bacteria, and requires a direct contact with the host cell. Furthermore, bacterial supernatant cannot induce the toxin effect, indicating that colibactin is directly injected into the host cells [7].

Infection of HeLa cells and non-transformed rat intestinal crypt IEC-6 cells with the *E. coli* strain DH10B hosting a bacterial artificial chromosome bearing the complete *pks*-island (BAC *pks*) induces DNA DSBs, as assessed by comet assays. This genotoxic stress triggers the classical DNA damage response: phosphorylation of H2AX and the checkpoint kinase CHK2, inactivation of the CDC25C phosphatase, and accumulation of the hyperphosphorylated form of CDC2, with the end result that cells undergo cell cycle arrest [7]. Non-transformed adherent cells that survive the acute bacterial infection display the hallmarks of senescence: expression of senescence-associated β-galactosidase (SA-β-Gal), formation of PML nuclear bodies, and formation of senescent-associated heterochromatic foci [63]. This phenotype is associated with production of ROS and secretion of pro-inflammatory cytokines, such as IL-6, IL-8, monocyte chemotactic protein (MCP)-1 and the matrix metallo-protease (MMP)-3. Conditioned medium from the *pks*-induced senescent cells is able to trigger DNA damage and SA-β-Gal activity in recipient cells, and can promote the growth of bystander tumor cells *in vitro* [63].

The initial discovery of colibactin was performed using the extra-intestinal pathogenic *E. coli* (ExPEC) strain IHE3034, isolated from newborn meningitides [7]. Detailed molecular epidemiological analysis of intestinal *E. coli* further demonstrated that the *pks* island is present with high frequency not only in a highly virulent subset of ExPEC isolates associated with multiple other virulence gene clusters and more prone to induce bacteremia [64,65], but also in long-term colonizers isolated from the gut of healthy infants followed for the first 18 months of life [66]. Even the probiotic *E. coli* strain Nissle 1917 carries the *pks* island [7].

## 4. Bacterial Genotoxins and Carcinogenic Potential

The mode of action of these three bacterial genotoxins in long-term colonizers [66] or pathogenic bacteria that can induce chronic asymptomatic infections (e.g., *Salmonella enterica*) [68] poses the question of whether they may contribute to the acquisition of genomic instability and promote cancer development in the context of chronic status carriers. Few studies have addressed this question.

### 4.1. Carcinogenesis Properties in Vitro

**Figure 5:**
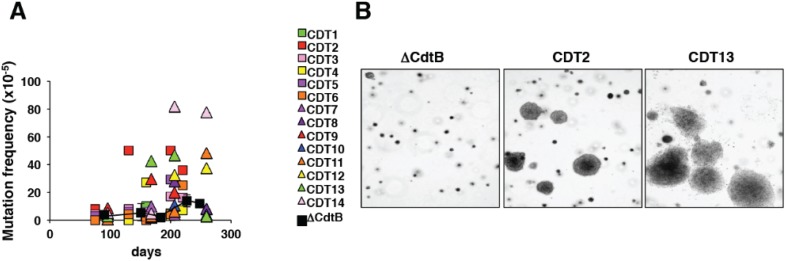
The carcinogenic potential of CDT *in vitro.* A total of 14 sub-lines of rat fibroblasts were selected by chronic exposure to sub-lethal doses of HhepCDT for a period of 220 days. As control, seven sub-lines were selected in the presence of an equal amount of an inactive mutant toxin. (**A**) Mutation frequency of a reporter gene in the 14 sub-lines of rat fibroblast exposed to the active CDT for the indicated periods of time. The mutation frequency of cells exposed to the inactive CDT (ΔCdtB) is presented as mean of all the seven sub-lines tested; (**B**) Chronic exposure to HhepCDT promotes anchorage-independent growth at 220 days post-selectin. Fifty thousand cells per sub-line were plated in 60 mm dishes in triplicate and colonies were visualized after culture for three weeks by staining with crystal violet. The panel shows representative micrographs of the colonies (4× magnification). The figure is adapted from [69].

Chronic exposure to sublethal doses of HhepCDT and HducCDT, which do not cause an acute cell cycle arrest, but promote limited DNA damage, induces increased mutation frequency and accumulation of chromatin aberrations in the absence of decreased viability or senescence, in cells of mesenchymal and epithelial origin (Figure 5A). The accumulation of genomic instability is associated with impaired activation of the DDR and failure to properly activate the checkpoint response upon exposure to genotoxic stress, and activation of pro-survival signals dependent on chronic activation of MAPK p38 [69]. These results suggest that the chronic intoxication promotes the survival and proliferation of cells carrying impaired mechanisms of detection and repair of DNA damage, possibly mimicking the stochastic selection process that occurs during cancer development *in vivo*. The increased genomic instability induced by the chronic intoxication further correlates with the acquisition of malignant traits such as the ability to grow in an anchorage-independent manner [69] (Figure 5B).

These data on chronic exposure to CDTs are paralleled by similar findings obtained with short term exposure of the hamster CHO cell line to *pks* positive *E. coli* at low multiplicity of infection (ranging from 5–20 bacteria per cell) for 4 h. In this experimental set-up, low levels of DNA damage are still detected 24 h post infection in actively cycling cells, well after the bacteria have been killed by addition of antibiotics, indicating that the DNA repair process was not completed. As consequence of the partial DNA repair, infected cells accumulated anaphase bridges and other chromosomal aberrations in approximately 7% of the chromosomes, detected as early as 72 h post-infection. Such aberrations are maintained in a proportion of cells up to 21 days post-infection. The chromosomal instability induced by infection with *pks* positive *E. coli* is further associated with enhanced rate of mutation frequency and increased ability of the cells to grow in an anchorage-independent manner [70].

### 4.2. Carcinogenesis Propertiesin in Vivo

The data presented in the previous paragraphs indicate that chronic exposure to CDTs or colibactin is carcinogenic *in vitro*. Epidemiological analyses support a possible role of these toxins in initiation/progression of gastro-intestinal tumors, since higher frequency of CDT [71] and colibactin [71,72,73] has been detected in mucosa-associated *E. coli* isolates from colorectal cancer (CRC) and Inflammatory Bowel Disease (IBD) patients compared to control subjects. To address experimentally this possibility, several studies on *in vivo* animal models have been performed, mostly using colibactin as genotoxin model.

Due to the association between IBD/CRC and a higher colonization with *pks* positive bacteria, Arthur and co-workers have used germ-free IL-10 to knock out mice (IL-10^−/−^, model for IBD) treated with the colon-specific carcinogen azoxymethane (AOM) as a model for colitis-associated CRC. These mice have a higher incidence of invasive adenocarcinoma when mono-colonised with the commensal *pks* positive *E. coli* strain NC101 compared to mice mono-colonised with an isogenic *pks* deficient strain or the control pks negative commensal bacterium *Enterococcus faecalis* [72]. Interestingly, the inflammatory score is equally high in all the mice, independently of the colonizing bacterium, while the presence of the *pks* island is the only requirement for tumor induction in this experimental set-up [72]. It is important to underline that the effect of the NC101 strain on tumor development is detected in mice highly susceptible to inflammatory conditions and pre-treated with a carcinogen, suggesting that the carcinogenic potential of this toxin requires a specific environment. This hypothesis is supported by an elegant series of experiments where the transcriptome of the *E. coli* NC101 strain was analyzed 2, 12, and 20 weeks after mono-colonization of three germ-free mouse models: IL-10^−/−^ (inflammation), AOM/IL-10^−/−^ (CRC and inflammation); and AOM/IL-10^−/−^; Rag2^−/−^ (no inflammation, no CRC, baseline reference) [74]. RNA seq analysis demonstrated that only few genes were differentially expressed in these three conditions at any time point tested. Specifically, expression of 66 *E. coli* genes was deregulated in the cancer microenvironment. Five *pks* island genes (*cblG*, *cblH*, *cblL*, *cblM* and *cblN*) were among the genes that presented the most significant change [74]. These data suggest that the tumor microenvironment may influence the gene expression of the intestinal microbiota or a subset of it, fuelling the pro-carcinogenic properties of bacterial genotoxins.

The tumor promoting effect of colibactin is linked to its capacity to trigger a senescence-associated secretory phenotype, resulting in a high production of growth factors that promote tumor growth [75]. This was demonstrated by injecting subcutaneously, in nude mice, HCT116 cells infected with the *pks* positive *E. coli* DH10B strain producing colibactin or an isogenic *pks* negative strain. Exposure to *pks* positive *E. coli* significantly increases tumor growth. This effect is sustained by the colibactin-induced cellular senescence, which promotes accumulation of the sumoylated form of the tumor suppressor protein p53. The accumulation of sumoylated p53 is dependent on the down-regulation of the Sentrin/SUMO-specific protease (SENP)-1, mediated by increased levels of the microRNA miR20a-5p. The relevance of these findings was confirmed in an *in vivo* model of CRC, which showed induction of senescence, accumulation of miR20a-5p, and reduced levels of SENP1 upon colonization of the mouse intestinal tract with the colibactin positive CCR20 strain, isolated from a human CRC specimen [75].

The role of CDTs in *in vivo* carcinogenesis is less studied, due to the lack of suitable models of chronic infection/colonization with CDT-producing bacteria. However, infection of mice homozygously deficient for p50 and heterozygous for p65 (referred to as 3X mice) with CDT-producing *C. jejuni* [76], promotes colonization of the gastro-intestinal tract and is associated with enhanced gastritis and hyperplasia at four months post-infection, compared to infection with an isogenic strain carrying a mutated *cdtB* gene, thus deficient for the genotoxic activity. Similarly, infection of IL-10^−/−^ mice with wild-type *H. hepaticus* or *H. cinaedi* enhances the inflammatory conditions [77,78,79]. This is associated with an altered pattern of cytokine response, since animals infected with the CDT-producing strain develop significantly higher production of Th1-associated immunoglobulin G2a (IgG2a), Th2-associated IgG1 and mucosal IgA [78,80], higher levels of pro-inflammatory mediators [79], and lower levels of the anti-inflammatory cytokine IL-10 [80]. Based on these results, it is likely that the presence of CDT promotes long-term infection and alters the host inflammatory response, and this may contribute to inflammation-associated carcinogenesis. This is supported by the demonstration that the presence of CDT is necessary for the development of hepatic dysplastic nodules 10 months after infection of A/JCr mice with *H. hepaticus* [81]. The pro-carcinogenic effect of HhepCDT is associated with enhanced hepatic transcription of pro-inflammatory (*TNF-α*, *IFN-γ* and *Cox-2*, *IL-6* and *TGF-*α) and anti-apoptotic (*Bcl-2* and *Bcl-X_L_*) *genes*, up-regulation of hepatic mRNA levels of components of the NF-κB pathway (p65 and p50), and increased hepatocyte proliferation [81].

## 5. DNA Damage induced by other Bacterial Effectors: *Helicobacter pylori*, *Pseudomonas aeruginosa* and Uropathogenic Strains of *Escherichia coli*

Beside the three known bacterial genotoxins, induction of DNA damage, which is not dependent on production of endogenous ROS, has been demonstrated for two other bacterial species: *H. pylori* and *P. aeruginosa*, and for another *E. coli* virulence factor present in uropathogenic strains, namely Usp.

*H. pylori* is a human pathogen which infects about 50% of the global population. The clinical outcome ranges from asymptomatic gastritis to peptic ulceration and gastric malignancy [82]. Despite the broad spread of *H. pylori* infection, only a small percentage of positive subjects develop gastric carcinoma [83]. This discrepancy is emblematic of the multifactoriality underlying the carcinogenesis process, where environmental factors, host-inflammatory genetic susceptibility, virulence factors, and variation of the bacterial strains should be globally considered [83]. For a detailed review on *H. pylori*, its virulence factors, and its role in carcinogenesis we refer the reader to recently published works [84,85,86]. Here we will focus on recent findings that are pertinent to the direct induction of DNA damage, in a ROS- and inflammation-independent manner. Toller *et al.* [10] demonstrated the accumulation of DNA DSBs in several *H. pylori* infected cell lines and primary gastric epithelial cells in a time- and dose-dependent manner. The factor responsible for this effect has not been yet identified, but the DNA damage induction requires direct contact of live bacteria with the host cells, it is independent of Cag Pathogenicity Island (PAI), and is not due to ROS production. The bacterial-induced DNA damage is associated with an ATM-dependent phosphorylation of H2AX (γ-H2AX), and formation of nuclear foci containing γ-H2AX, the mediator of DNA damage checkpoint protein 1 (MDC1), and the p53-binding protein 1 (53BP1). In conclusion *H. pylori*, together with genotoxins expressing bacteria, is one of the few cases currently known of bacteria directly inducing DNA damage of host cell nuclear DNA.

Another bacterium recently found to possibly induce directly DNA damage is *P. aeruginosa*, an ubiquitous Gram-negative bacterium associated with nosocomial diseases, causing severe infections in immunocompromised patients, in patients with cystic fibrosis, or with severe burn wounds [87]. Initial observations reported induction of DNA breaks and increased activity of OGG1, an enzyme belonging to the Base Excision Repair system, in lung epithelial cells infected by *P. aeruginosa* strain PAO1 [88]. The increased OGG1 activity was also detected in an *in vivo* mouse model of *P. aeruginosa* infection. Subsequently, Elsen and co-workers [8] demonstrated that infection with different *P. aeruginosa* strains causes DNA strand breaks and phosphorylation of histone H2AX and its co-localization with the 53BP1 protein in terminally differentiated macrophages, and in actively proliferating lung cancer epithelial cells at 1.5 h–2.5 h post-infection. This effect is dependent on activation of the ATM kinase, and partially on the phosphorylation of the MAP kinase (MAPK) c-Jun. The genotoxic activity is abolished in isogenic strains where the gene encoding for the ExoS toxin has been deleted [8]. ExoS is a bacterial effector that is injected into the host cytoplasm through the type III secretion system (T3SS) [89], and transported from the plasma membrane through endosomes, the TGN and the ER to a perinuclear localization [90]. This virulence factor has no nuclease activity, but is characterized by a GTPase-activating protein (GAP) activity and an ADP ribosyltransferase (ADP-RT) domain. The latter is the enzymatic activity involved in the formation of γH2AX foci upon *P. aeruginosa* infection, but the target(s) of the ExoS-dependent ADP ribosylation is still unknown [8]. It is noteworthy that the authors did not formally exclude that the damage was indirectly caused by ROS production, since no anti-oxidant agents were used in the experimental set-up. However, an isogenic strain, mutant for the T3SS, but expressing the lipopolysaccharide (LPS), known to trigger ROS production, was unable to induce H2AX phosphorylation [8].

The vast majority of uropathogenic *E. coli* strains isolated from pyelonephritis, prostatitis, and bacteremia of the urinary tract, harbour the uropathogenic-specific gene, *usp* [91], encoded within a small pathogenicity island together with 3 downstream open reading frames, *imu1–3.* The Usp C-terminal domain shares a 40%–45% identity with DNase-like colicins and pyocins, collectively known as bacteriocins, antimicrobial factors known to inhibit the growth of closely related bacterial strain(s) [92], while Imu 1–3 exhibit similarity with immunity proteins protecting colicin producers strains against their own bacteriocins [9]. Nipic *et al.* demonstrated that Usp is a novel genotoxin active against eukaryotic cells, which does not exert any antimicrobial activity [9]. Purified Usp cleaves linearized naked DNA *in vitro* and causes DNA fragmentation in mammalian cells, as assessed by comet assay. Interestingly, the DNase activity of purified Usp on a linearized plasmid is less potent than that exhibited by the bacteriocin colicin E7. This effect is reminiscent of the lower potency of the CdtB subunit of CDTs when compared to the activity exerted by the mammalian DNase I [19]. The genotoxic activity of this effector on mammalian cells is greatly enhanced when Usp is delivered together with the Imu2 protein. The result of intoxication with purified Usp-Imu2 or infection with *usp* and *imu1–3 E. coli* positive strains is induction of cell rounding, collapse of the actin cytoskeleton, and induction of caspase 3/7-mediated apoptosis. It will be very interesting to evaluate whether short term exposure to purified Usp-Imu2 or infection with *usp* and *imu1–3* positive *E. coli* strains activate the classical DDR, as previously shown for CDTs, the typhoid toxin, and colibactin.

## 6. What is the Role of Bacterial DNA-Damaging Effectors?

Evidences discussed in previous paragraphs clearly indicate that under specific circumstances the presence of colibactin and CDT are carcinogenic. Furthermore, *H. pylori*, the first bacterium recognised as a human carcinogen by the World Health Organization [93], also possesses direct genotoxic activity.

However, these findings pose several questions: What is the role of these unusual genotoxins/genotoxic activities in the physiology of the host-microbe interaction? Why have bacterial toxins with a low DNase activity been maintained and possibly horizontally transferred?

One clue may come from the observation that the anti-inflammatory effect of the probiotic *E. coli* strain Nissle 1917 is strictly dependent on the colibactin activity. Deletion of the *cblA* gene abolishes the capacity of this bacterium to induce DNA damage in intestinal crypt IEC-6 cells infected for 4 h at a multiplicity of infection (MOI) of 100:1. However, the loss of the genotoxic effect completely neutralised the anti-inflammatory properties of the Nissle strain 1917 in two models of colitis: the Dextran Sodium Sulfate (DSS)-induced colitis in rats and a T-cell dependent model of chronic colitis induced by the adoptive transfer of naive CD4^+^ CD45RB^high^ T cells in immunocompromised SCID mice [94].

Similarly, the presence of the typhoid toxin in a *S.* Typhimurium strain reduces the mortality rate of the host, promotes chronic asymptomatic status carriers, and decreases the inflammatory response in the intestine of infected immunocompetent sv129 mice (Frisan, unpublished observation) [95].

Thus, it is likely that the primary effect of the bacterial effectors that cause DNA damage is to subtly modulate the host immune response, favouring colonization and consequently bacterial spread to new hosts, rather than enhancing the virulence traits of the infection process. The carcinogenic properties could therefore be an “accident” due to a sum of factors (e.g., inflammatory predisposition, susceptibility to cancer due to genetic or environmental factors, and co-infections), as shown for other micro-organisms associated with cancer, such as the Human Papilloma Virus [96].

Further studies and the development of novel models that mimic chronic infection or asymptomatic status carriers in immunocompetent hosts are required to provide conclusive answers to these questions.
